# Predictors of participant compliance with ecological momentary assessment among individuals with chronic pain who are using cannabis and opioids

**DOI:** 10.1016/j.invent.2024.100784

**Published:** 2024-10-26

**Authors:** Jungin Joo, Janardan Devkota, Bryant M. Stone, Kelly E. Dunn, Vadim Zipunnikov, Ryan Vandrey, Patrick H. Finan, Johannes Thrul

**Affiliations:** a Department of Digital Health, SAIHST, Sungkyunkwan University, Seoul, Republic of Korea; b Department of Mental Health, Johns Hopkins Bloomberg School of Public Health, Baltimore, MD, USA; c Department of Psychiatry & Behavioral Sciences, Johns Hopkins University School of Medicine, Baltimore, MD, USA; d Department of Biostatistics, Johns Hopkins Bloomberg School of Public Health, Johns Hopkins University, Baltimore, MD, USA; e Department of Anesthesiology, School of Medicine, University of Virginia, Charlottesville, VA, USA; f Sidney Kimmel Comprehensive Cancer Center at Johns Hopkins, Baltimore, MD, USA; g Centre for Alcohol Policy Research, La Trobe University, Melbourne, Australia

**Keywords:** Ecological momentary assessment, Cannabis, Opioid, Patterns, Chronic pain, Compliance

## Abstract

**Background:**

Cannabis may be an alternative or adjunct to opioid therapy for chronic pain. However, there are limited data on patterns of opioid medication and medical cannabis use. The current study investigated the feasibility of using Ecological Momentary Assessment (EMA) to assess patterns of prescription opioids and medical cannabis among individuals experiencing chronic pain.

**Method:**

The study included 133 participants recruited online. Participants were 42.6 (SD = 13.9) years old on average and the majority were men (57.9 %) and Non-Hispanic White (63.2 %). Participants completed a baseline assessment, followed by 30 days of EMA data collection with four randomly prompted past-hour surveys and one daily diary per day, and a follow-up survey that assessed perceived EMA burden. Simple and multivariable linear regression models were estimated to investigate participant predictors of the proportion of EMA surveys completed (past-hour surveys and daily diaries in separate models).

**Results:**

Compliance rates for EMA prompts were 89.7 % for daily diaries and 63.3 % for past-hour surveys. In multivariable regression, participants holding a graduate degree completed a lower proportion of daily diaries (*b* = −0.109, *SE* = 0.052, *p* < .05) and past-hour surveys (*b* = −0.148, *SE* = 0.071, *p* < .05), compared to those with less than a 4-year degree. Participants completing a higher proportion of daily diaries reported greater ease of use at follow-up (*b* = 0.050, *SE* = 0.022, *p* < .05) and those completing a higher proportion of past-hour surveys desired higher rewards (*b* = 0.066, *SE* = 0.033, *p* < .05).

**Conclusions:**

Study results confirm the feasibility of using EMA methods to assess patterns of prescription opioids and medical cannabis among individuals experiencing chronic pain.

## Highlights

• We collected EMA data on opioid and cannabis use in 133 patients with chronic pain.
• Compliance was 89.7 % for daily diaries and 63.3 % for past-hour surveys.
• Higher education was associated with lower EMA compliance.
• Those with higher compliance reported greater ease of use and desired more rewards.

## 1 Introduction

Chronic pain affects 51.6 million U.S. citizens, accounting for 20.9 % of American adults in 2021 (Rikard et al., 2023). Among patients with chronic pain, 22 % report using prescription opioids in the past three months (Dahlhamer et al., 2021). Moreover, a review study indicated that among chronic pain patients up to 29 % may misuse prescription opioids and up to 12 % may meet criteria for opioid addiction (Vowles et al., 2015). Despite a recent decline in opioid prescriptions for chronic non-cancer pain, they remain a mainstay of pain management for a variety of patients, particularly those with cancer pain (Bandara et al., 2022).

Clinical practice guidelines advocate for non-opioid pharmacologic therapy for chronic pain to reduce concerns regarding opioid misuse (Dowell et al., 2016). Cannabis may be an alternative or adjunct to opioid therapy for chronic pain, and chronic pain is the single most frequent reason for medical cannabis use (Boehnke et al., 2019). In adults with chronic pain, individuals treated with cannabis or cannabinoids experience a clinically significant reduction in pain symptoms (NASEM, 2017). Furthermore, a recent study using data of the New York State medical cannabis program reported significantly greater reductions in opioid dosage among patients with chronic pain who received medical cannabis for >30 days compared to those who received medical cannabis for 30 days or fewer (Nguyen et al., 2023).

However, there are limited data on the patterns of opioid medication and medical cannabis from studies using intensive longitudinal designs. While prospective studies can elucidate long-term changes in cannabis and opioid use, they cannot speak to fine-grained patterns of cannabis and opioid use on a given day. Retrospective surveys may be limited by recall bias. Intensive longitudinal data are needed to investigate patterns of opioid medication and medical cannabis. In addition, this intersection of populations experiencing both substance use and chronic pain often presents complex health challenges, including elevated risks for adverse outcomes, which makes it crucial to study (Dassieu et al., 2019).

Ecological Momentary Assessment (EMA) is a data collection method that involves repeated sampling of participants' behaviors and experiences in real time over a day and in participants' natural environments. EMA aims to minimize recall bias, maximize ecological validity, and allow the study of micro processes that influence behavior in real-world contexts (Shiffman et al., 2008). EMA methods are well-suited to studying substance use and have seen wide adoption in this area of research. Substance use lends itself to event-based recording, making EMA a useful method for tracking its frequency and distribution over time (Shiffman, 2009).

The quality of EMA data is reliant on participants completing repeated assessments with high compliance. EMA methodology has demonstrated completion rates of the surveys ranging from 75 % to 86 % in EMA studies of chronic pain or substance use (Jones et al., 2019; May et al., 2018; Ono et al., 2019). One study in particular found that age and study day each independently influenced completion rates, with younger participants having lower rates and completion rates declining over time (Ono et al., 2019). However, beyond age and gender, these previous reviews did not investigate the associations between sociodemographic variables and EMA compliance, which is a limitation in the existing literature.

A recent study our group conducted used EMA methodology to investigate the co-use patterns of opioids and/or cannabis in chronic pain (Anderson Goodell et al., 2021; Mun et al., 2022). Participants completed EMA assessments over a period of 30 days and received 4 random prompts distributed over the course of the day and one daily diary. Participants completed 70 % of past-hour surveys and 92 % of daily diaries (Anderson Goodell et al., 2021). However, this previous study had several limitations, including a small sample size of 46 participants, who were predominantly female (78 %) and Non-Hispanic White (85 %), and replication of these findings with a larger and more diverse sample is needed. Moreover, our previous study did not assess participant perceived burden with EMA survey completion (Eisele et al., 2022).

To address the limitations in the existing literature, the present study aims to investigate the feasibility and acceptability of using EMA methods to assess patterns of prescription opioid and medical cannabis use among a larger and more diverse sample of individuals experiencing chronic pain. Moreover, the current study explores the perceived EMA burden among participants and its association with participant compliance with EMA survey completion.

## 2 Methods

### 2.1 Study overview

The study consisted of online surveys, smartphone-based 30-day EMA data collection including daily dairies and past-hour surveys, and an assessment of EMA burden after EMA data collection was completed. All study procedures were approved by the Institutional Review Board of the Johns Hopkins Bloomberg School of Public Health (Protocol: IRB00009327).

### 2.2 Participants

Recruitment was conducted using Facebook, Twitter, and Reddit advertisements. The research team also made organic posts with recruitment information in several subreddits (e.g., r/ChronicPain) as well as Facebook groups focusing on chronic pain. Moreover, recruitment was supported by the Colorado-based Realm of Caring Foundation focused on medical cannabis, which shared recruitment information on social media to their over 100,000 followers. All advertisements and posts included links to an online eligibility questionnaire and informed consent form (Anderson Goodell et al., 2021; Mun et al., 2022; Thrul et al., 2024). Participants were recruited from February 2022 to November 2022 from US states Alaska, California, Colorado, Illinois, Maine, Massachusetts, Michigan, Nevada, Oregon, Vermont, Washington, or Washington, D.C. These jurisdictions had legalized adult access to cannabis for both medicinal and non-medicinal purposes at the time of data collection.

Study participants were at least 18 years old and had to meet all of the following criteria: 1) currently have a prescription for opioids for pain symptoms; 2) currently used opioids (past seven days); 3) had recently (past 30 days) received a recommendation for medical cannabis or started using cannabis; 4) reported a chronic pain disorder; 5) reported pain at a minimum of 3/10 pain intensity on ≥10 days per month for the past 3 months or longer; 6) had an iPhone or Android smartphone; 7) lived in one of the previously mentioned US states in which cannabis was legal for recreational use.

Participants were excluded from the study if they self-reported having a severe psychiatric disorder (schizophrenia, psychosis, or dementia) that was deemed by the study team to potentially interfere with completing study procedures.

### 2.3 Procedure

Participants who completed the initial screening, consent, and ID verification procedures were invited for a baseline survey hosted on Qualtrics to assess sociodemographic characteristics, substance use, and chronic pain history. Participants were asked to identify the medications they had used in the past month from a list of short-acting, long-acting, oral, or non-oral opioid medications and cannabis products (i.e., flower, oil, concentrates, edibles, topicals), and prescription medications. After the baseline survey was completed, participants enrolled in the EMA phase of the study. EMA data were collected using the MetricWire smartphone application (available for both Android and iOS). We provided detailed instructions to participants on installing and registering the app, creating a password, and enabling push notifications during the installation process. These instructions were included in the email that contained the app installation link. Random EMA surveys were prompted 4 times per day within pre-determined time windows (8 AM–12 PM, 12–4 PM, 4–8 PM, 8–11 PM). These surveys were open for 1 h after they were prompted. Past-hour surveys assessed mood, opioid, cannabis, and other substance use, pain symptoms, potential pain relief, and pain catastrophizing, and situational characteristics (e.g., location, social context, activities) during the previous hour. Daily diaries were prompted between 10 and 11 AM and open for 12 h to complete. Daily diaries assessed extent of use of cannabis, opioids, and other substances, pain levels, and a number of quality-of-life measures on the entire previous day.

During the EMA phase, participants were provided support to resolve any issue on the application by instant messenger on the EMA app, text message, and/or email. The most frequent problems requiring troubleshooting were related to app registration and notification issues. For example, 3 participants did not follow the installation and registration link but instead installed the app directly from the app store and registered with a different email address. Additionally, 7 participants experienced issues with not receiving EMA survey notifications in the app, commonly due to low disk space on their device or low phone battery/low-battery mode. Participants were regularly updated on their progress by text message and/or email on days 3, 4, 14, 21, and 30 of the EMA study periods and were informed about their cumulative EMA survey completion rate. At the end of the 30-day EMA phase, participants completed a follow-up survey on Qualtrics.

Participants received $2 for completing at least one EMA survey on a given day (maximum $60 for 30 days), plus a bonus of $60 if they achieved a 75 % or greater overall EMA compliance rate at the end of the data collection period. The incentives were provided by Amazon electronic gift cards, which were emailed to participants.

### 2.4 Measures

#### 2.4.1 Outcome

The main outcome of the current study was EMA feasibility, which was operationalized as compliance. Compliance by time of day and day of the week was operationalized as a binary outcome of survey completed vs. missed. Compliance over the entire 30-day EMA study period was operationalized as the proportion of surveys completed to surveys prompted for past-hour surveys and daily diaries.

#### 2.4.2 Baseline survey

The baseline survey assessed sociodemographic information, including age, sex, race/ethnicity, education, the number of days of alcohol use in a typical week (dichotomized to any vs. none), as well as number of days of opioid medication use and medical cannabis use in the past 30 days.

To assess the severity of chronic pain at baseline, the Graded Chronic Pain Scale-Revised (GCPS-R) was administered (Von Korff et al., 2020). The GCPS-R measures pain intensity and the related disability with daily activities and allows classifying participants into 4 chronic pain severity grades: Grade 0: Chronic Pain Absent; Grade 1: Mild Chronic Pain; Grade 2: Bothersome Chronic Pain; and Grade 3: High Impact Chronic Pain.

#### 2.4.3 Follow-up survey

Participants' burden with EMA assessments was assessed at the end of the 30-day EMA data collection period with an instrument that was slightly adapted from a previous study (Eisele et al., 2022). The scale contained 19 items in five thematic subscales including burden (9 items; e.g., “I found it stressful to use the app.”), ease of use (3 items; e.g., “The questionnaires on the phone were easy to complete.”), instruction (3 items; e.g., “The training I received at the beginning of the study was adequate to use the app for 30 days.”), reward (1 item; “How much money do you find appropriate to receive for the participation in this study?”), and careless responding (3 items; e.g., “I didn't pay much attention to what the questions actually meant.”). Responses were recorded on a Likert scale from 1 (“not at all”) to 7 (“very much”). One exception was the reward item, where participants entered their desired incentive amount in dollars. Due to the large standard deviation, positive skewness, and high kurtosis, the reward subscale of the EMA Burden instrument was normalized using a logarithmic transformation.

#### 2.4.4 Statistical analyses

We presented a descriptive summary of sociodemographic characteristics of study participants, as well as the summary of EMA reporting and compliance. Past-hour survey completion by time of day and day of the week (binary outcome: survey completed vs. missed) were investigated using logistic regression. Daily diaries were only analyzed by day of the week due to time of day not being applicable. Simple and multivariable linear regression models were estimated to predict EMA compliance over the entire 30-day EMA study period in two separate models for past-hour surveys and daily diaries. Predictors included participant baseline characteristics as well as EMA burden subscales. Multivariable regressions included sociodemographic variables by default (age, sex, race/ethnicity, and education). Other predictors were selected based on significance in simple regression models. All analyses were conducted using R 4.3.1 and R Studio Version 2023.0.9.1 + 494.

## 3 Results

### 3.1 Overall study participant flow and sample description

**Fig. 1:**
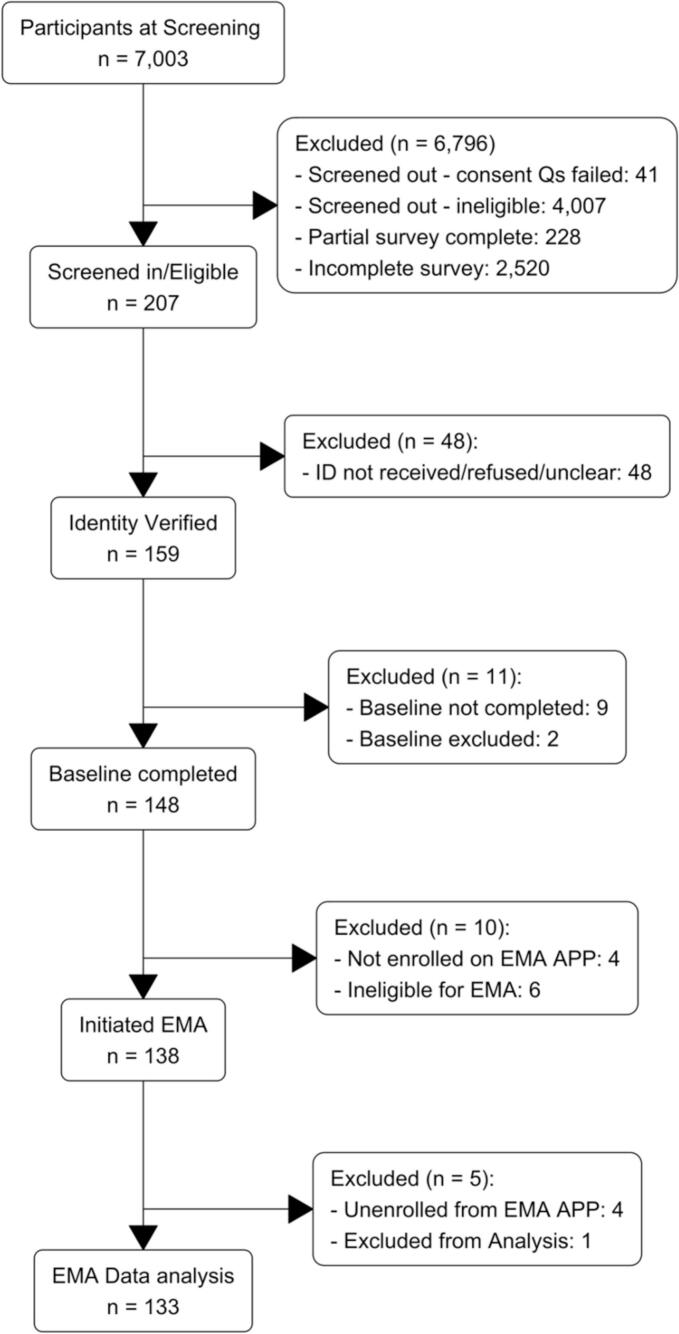
Participant flow through phases of study using smartphone-based EMA.

The participant flow through the study is shown in Fig. 1. Of 207 eligible participants, 159 (77 %) verified their identity, 148 (71 %) completed the baseline survey and were invited to the EMA phase, and 138 (67 %) initiated the EMA phase. Of these, 4 (2 %) participants requested to be unenrolled over the course of the EMA data collection phase and 1 participant was excluded due to problems with tracking the correct opioid medication used during the EMA phase. The final analytical sample included data of 133 participants who completed the entire 30-day EMA phase. The mean age of study participants was 42.6 (SD = 13.9), the majority were men (57.9 %), and Non-Hispanic White (63.2 %). All participant baseline characteristics are displayed in Table 1.

**Table 1:** Sample baseline characteristics (*N* = 133).

Characteristic	N (%) or MEAN (SD)
Age	42.6 (13.9 %)
Sex	
Female	56 (42.1 %)
Male	77 (57.9 %)
Race/Ethnicity	
NH African American or Black	30 (22.6 %)
NH Asian	2 (1.5 %)
NH White	84 (63.2 %)
Hispanic or Latino	13 (9.8 %)
NH Multiracial	4 (3.0 %)
Education	
<4-year degree	57 (42.9 %)
4-year degree	54 (40.6 %)
Graduate degree	22 (16.5 %)
Any alcohol use in a typical week ^1^	
No	56 (42.4 %)
Yes	76 (57.6 %)
Opioid and cannabis use	
Opioid use over past 30 days (mean (SD))	19.3 (9.1)
Cannabis use over past 30 days (mean (SD))	14.6 (11.8)
Graded Chronic Pain Scale-Revised	
Grade 0	15 (11.3 %)
Grade 1 & 2	38 (28.6 %)
Grade 3	80 (60.2 %)

Note: NH = Non-Hispanic; ^1^N = 132 due to a missing value for one participant.

### 3.2 EMA assessments

A total of 19,941 EMA surveys were prompted over the EMA data collection period, consisting of daily diaries (*n* = 3990) and past-hour surveys (*n* = 15,951). Nine submissions of past-hour surveys were removed in the data cleaning stage due to multiple survey submissions to the same trigger, likely due to app malfunction. Table 2 summarizes the volume of surveys distributed, and the rate of survey completion, across EMA survey types.

**Table 2:** Summary of EMA reporting and compliance (N = 13,668 surveys).

Daily diaries (*n* = 3577)	
Completed daily diaries / Expected total diaries	3577/3990 (89.7 %)
Total daily diaries per participant (*m* ± SD; range)	27.1 ± 6.7; 1–30
Participants completing 100 % of prompted daily diaries	78/133 (58.7 %)
Participants completing at least 90 % of prompted daily diaries	112/133 (84.2 %)

Past-hour surveys (*n* = 10,091)	

Completed past-hour surveys / Total sent random prompts	10,091/15,951 (63.3 %)
Total past-hour surveys per participant (*m* ± SD; range)	78.2 ± 30.2: 1–120
Participants reporting 1+ past-hour survey on each of the 30 days	73/133 (54.9 %)
Participants completing at least 75 % of prompted past-hour surveys	55/133 (41.4 %)
Observations per participant day (days w/ 1+ completed survey) (*m* ± SD)	3.0 ± 1.0

SD = standard deviation.

For daily diaries, out of the 3990 total prompted diaries, 3577 were completed, resulting in a compliance rate of 89.7 %. The number of participants who completed 100 % of daily diaries was 78 (58.7 %), and 112 (84.2 %) completed at least 90 % of daily diaries.

Regarding past-hour surveys, a total of 15,951 surveys were prompted and 10,091 were completed, resulting in a compliance rate of 63.3 %. The number of participants who completed at least 75 % of the past-hour survey prompts was 55 (41.4 %). On days with any past-hour survey completion, the mean number of completed past-hour surveys was 3.0 (SD = 1.0).

During the 30-day EMA study period, 86 out of 133 participants submitted only one of the 4 past-hour surveys in a day, with an average of 4.2 days (median of 3 days, IQR: 2–5.8 days), ranging from a minimum of 1 day to a maximum of 18 days, and accounting for 14 % of the total study days. The average delay time from the issuance of the randomly prompted past-hour survey notification to the start of the response was approximately 16 min (mean) and 10 min (median).

### 3.3 EMA burden assessment at follow-up

Among 133 participants, 131 completed the follow-up survey, while 2 did not. The average completion rates for daily diaries were 90.7 % for the 131 participants and 21.7 % for the 2 who did not complete the follow-up survey. For past-hour surveys, the rates were 64.0 % and 10.8 %, respectively.

Participants reported a mean burden score of 2.6 (SD = 1.2), a mean ease of use score of 5.3 (SD = 1.2), a mean instruction score of 5.6 (SD = 1.4), an average desired reward of $178 (SD = 98.4) for the 30 days, and a mean score for careless responding of 1.4 (SD = 0.7).

Individual items for these subscales were recorded on a Likert scale from 1 to 7, with higher scores indicating greater endorsement of the respective subscale.

### 3.4 Associations between participant characteristics and compliance outcomes

#### 3.4.1 Outcome: Past-hour survey completion by time of day and day of the week

There were no significant differences in past-hour survey completion by time of day, with one exception: Compared to past-hour surveys during the first time window of the day from 8 AM–12 PM (65.4 % completion rate), those surveys prompted during the last time window of the day from 8 to 11 PM (57.0 % completion rate) were less frequently completed (OR = 0.70, 95 % CI: 0.64–0.77, *p* < .001). Day of the week was not significantly associated with likelihood of past-hour survey and daily diary completion.

#### 3.4.2 Outcome: Daily diary completion over the entire 30-day EMA study period

None of the participant baseline characteristics were associated with the proportion of daily diaries completed in simple regression analyses (Table 3).

**Table 3:** Results of simple and multivariable linear regression analyses of EMA survey compliance over the 30-day study period (N = 133).

	Daily diaries		Past-hour surveys	
	Simple regression	Multivariable regression	Simple regression	Multivariable regression
	Proportion of surveys b (SE)	Proportion of surveys b (SE)	Proportion of surveys b (SE)	Proportion of surveys b (SE)
Baseline characteristics				
Sex				
Male	Ref	Ref	Ref	Ref
Female	−0.070 (0.041)	−0.041 (0.037)	−0.061 (0.048)	−0.063 (0.047)

Age				
Younger than 40	Ref	Ref	Ref	Ref
40 years or older	0.057 (0.041)	0.043 (0.037)	0.047 (0.047)	0.027 (0.045)

Race				
Caucasian	Ref	Ref	Ref	Ref
African American or Black	−0.037 (0.051)	−0.038 (0.045)	−0.049 (0.058)	−0.004 (0.058)
Hispanic or Latino & Others	0.017 (0.060)	−0.012 (0.052)	0.046 (0.069)	0.026 (0.062)

Education				
<4-year degree	Ref	Ref	Ref	Ref
4-year degree	0.007 (0.045)	−0.060 (0.039)	0.006 (0.050)	−0.030 (0.053)
Graduate degree	−0.096 (0.059)	−0.109 (0.052) ⁎	−0.182 (0.067) ⁎⁎	−0.148 (0.071) ⁎

Any alcohol use in a typical week				
No	Ref		Ref	Ref
Yes	−0.019 (0.042)		−0.110 (0.047) ⁎	−0.089 (0.053)

Opioid				
Number of opioid medication use days^1^	0.004 (0.002)		0.005 (0.003) ⁎	0.002 (0.003)

Cannabis				
Number of cannabis use days^1^	0.001 (0.002)		0.003 (0.002)	

Graded Chronic Pain Scale				
Grade 0	Ref		Ref	
Grade 1 & 2	−0.006 (0.072)		0.055 (0.083)	
Grade 3	0.038 (0.067)		0.065 (0.077)	

EMA Burden^2^				
Burden	−0.033 (0.016) ⁎	0.023 (0.019)	−0.029 (0.019)	
Ease of use	0.068 (0.015) ⁎⁎⁎	0.050 (0.022) ⁎	0.082 (0.018) ⁎⁎⁎	0.037 (0.023)
Instructions	0.040 (0.014) ⁎⁎	0.020 (0.015)	0.057 (0.016) ⁎⁎⁎	0.016 (0.020)
Reward	0.054 (0.026) ⁎	0.035 (0.028)	0.084 (0.032) ⁎	0.066 (0.033) ⁎
Careless responding	−0.028 (0.026)		−0.062 (0.031) ⁎	−0.040 (0.030)

Note: SE = standard error; CI = confidence interval.

⁎ *p* < .05;

⁎⁎ *p* < .01;

⁎⁎⁎ *p* < .001;

1 Past 30 days;

2 N = 131 due to two follow-up losses.

Participants who reported less burden (*b* = −0.033, *SE* = 0.016, *p* < .05), perceived greater ease of use (*b* = 0.068, *SE* = 0.015, *p* < .001), gave higher ratings to EMA instructions (*b* = 0.040, *SE* = 0.014, *p* < .01), and desired higher rewards (*b* = 0.054, *SE* = 0.026, *p* < .05) completed a higher proportion of daily diaries in simple regression analyses.

In the multivariable regression analysis (Table 3), participants holding a graduate degree completed a lower proportion daily diaries compared to those with less than a 4-year degree (*b* = −0.109, *SE* = 0.052, *p* < .05). There was a positive association between reported ease of use at follow-up and proportion of daily diaries completed (*b* = 0.050, *SE* = 0.022, *p* < .05). The R-squared and adjusted R-squared for daily diaries were 19.1 % and 12.3 %, respectively.

#### 3.4.3 Outcome: Past-hour survey completion over the entire 30-day EMA study period

In simple regression analyses (Table 3), participants holding a graduate degree completed a lower proportion of past-hour surveys compared to those with less than a 4-year degree (*b* = −0.182, *SE* = 0.067, *p* < .01). The mean percentage of past-hour surveys completed was 66.0 % for those with less than a 4-year degree, 66.6 % for those with a 4-year degree, and 47.8 % for those with a graduate degree, respectively. Moreover, those who reported using any alcohol in a typical week completed a lower proportion of past-hour surveys (*b* = −0.110, *SE* = 0.047, *p* < .05), and baseline number of days of opioid use had a positive association with proportion of past-hour surveys completed (*b* = 0.005, *SE* = 0.003, *p* < .05).

Participants who perceived greater ease of use (*b* = 0.082, *SE* = 0.018, *p* < .001), gave higher ratings to EMA instructions (*b* = 0.057, *SE* = 0.016, *p* < .001), desired higher rewards (b = 0.084, *SE* = 0.032, *p* < .05), and reported less careless responding (*b* = −0.062, *SE* = 0.031, *p* < .05) completed a higher proportion of past-hour surveys in simple regression analyses.

In the multivariable regression analysis (Table 3), participants holding a graduate degree completed a lower proportion of past-hour surveys compared to those with less than a 4-year degree (*b* = −0.148, *SE* = 0.071, *p* < .05). Moreover, participants who desired higher rewards at follow-up completed a higher proportion of past-hour surveys (*b* = 0.066, *SE* = 0.033, *p* < .05). No other baseline or EMA burden variables were significantly associated with proportion of past-hour survey completion in the multivariable regression model. The R-squared and adjusted R-squared values for past hour surveys were 26.8 % and 19.3 %, respectively.

## 4 Discussion

The aim of current study was to investigate the feasibility of using EMA to assess patterns of prescription opioid and medical cannabis use among individuals experiencing chronic pain and to identify participant predictors of EMA compliance. The compliance rates for EMA surveys in this study were 89.7 % in daily diaries and 63.3 % in past-hour surveys. In multivariable regression models, participant education (graduate degree associated with lower compliance for both daily diaries and past-hour surveys), the ease of use subscale (participants with higher daily diary compliance reported greater ease of use at follow-up) and the rewards item of the EMA burden scale (participant with higher past-hour survey compliance desired higher rewards) were associated with EMA compliance.

Compared to a previous review study using EMA methodology in chronic pain research, which reported an average completion rate of 86 % across 32 projects (May et al., 2018), our study achieved higher completion rates for daily diaries but lower completion rates for past-hour surveys. In the current study, 5 EMA surveys were prompted each day, which is similar to previous studies (mean of 5.9 surveys, median of 5.0 surveys). However, it should be noted that the current study collected EMA data over a period of 30 days, while the review reported an average study duration of 23.4 days (median of 14.0 days) (May et al., 2018). Moreover, most studies included in the review used palmtop computers, while only a minority used smartphones (14.5 %), and compliance rates for those smartphone studies ranged from 69.8 % to 89.7 % (May et al., 2018). The average smartphone user in the US currently receives 46 app push notifications per day (Dogtiev, 2024), which may make it more difficult for current EMA studies to achieve high compliance due to competition of study prompts with other phone notifications. While there is no widely accepted gold standard or justification for specific EMA compliance cutoffs that indicate high data quality, a target of 80 % has been discussed in the literature (Jones et al., 2019). However, despite the lack of a clear gold standard, high compliance rates have importance of for generalizability of EMA data (Shiffman et al., 2008). Therefore, improving participant compliance with smartphone-based EMA across all types of EMA surveys in the context of potential saturation of smartphone notifications should be a priority for the field.

Participants in our study had lower compliance with past-hour surveys compared to daily diaries. This is not surprising considering that daily diaries were prompted only once per day and were available to be completed for 12 h following the prompt on the study app. In contrast, the past-hour surveys were prompted 4 times per day and were only available to be completed for 1 h. Moreover, when investigating time of day effects on past-hour survey completion, we found that surveys prompted late in the day (8–11 PM) had significantly lower completion rates compared to those prompted early in the day (8 AM-12 PM). This may be due to the late survey window overlapping with sleep time for some participants and future studies may want to allow participants to tailor the time window of assessments late in the day with this in mind.

In our previous study using the same design, participants achieved higher compliance, completing 70 % of past-hour surveys and 92 % of daily diaries (Anderson Goodell et al., 2021). However, this previous study had a different and more homogenous sample composition (e.g., predominantly female (78 %) and Non-Hispanic White (85 %)), while the sample was more diverse with regards to gender (42 % female) and race/ethnicity (63 % Non-Hispanic White). In sum, both our previous study (Anderson Goodell et al., 2021) and the current study consistently show lower completion rates for past-hour surveys, therefore, especially compliance with these types of EMA surveys should be improved in future studies. For example, future studies may want to experimentally manipulate the number of survey prompts delivered every day to investigate if fewer prompts might improve compliance.

Among all participant characteristics, only education showed a significant association with EMA compliance for both past-hour surveys and daily diaries in multivariable regression analyses, such that individuals with a graduate degree had lower compliance compared to those who had less than a 4-year college degree. Potential explanations for this finding could be that individuals with higher education have more demanding work obligations that may make them miss study notifications. Moreover, individuals with higher education may have more disposable income and the study incentives for high compliance may not be as valuable to these participants. A recent survey also indicated that individuals with higher levels of education may be less dependent on smartphones for online access compared to those with lower levels of education, as they often have both a smartphone and home broadband (PEW Research Center, 2024). This could result in reduced smartphone usage in favor of other devices, potentially leading to missed notification.

This finding may have clinical significance, suggesting that tailored assessments may be needed for this group. For instance, improving the app interface or providing more flexible survey completion options could increase compliance among those with graduate degrees. Addressing these educational disparities is crucial, as higher compliance is often associated with better self-monitoring and health management. Based on our findings, future EMA studies in the area of chronic pain and substance use research may want to especially focus on increasing compliance rates among those with higher education, as this could enhance data accuracy and ultimately improve clinical outcomes for this group.

The current study also investigated associations between participant EMA compliance and an EMA burden instrument at follow up that included subscales on burden, ease of use, instruction, rewards, and careless responding (Eisele et al., 2022). In multivariable regression analyses, only the ease of use subscale was associated with higher compliance for daily diaries and only the rewards item was associated with higher compliance in past-hour surveys. These findings underline the importance of designing EMA data collection approaches that are easy to adhere to for participants and suggest that participants who completed more EMA surveys, especially in the more frequent and likely more burdensome past-hour assessments, may require higher incentives at the end of the study. As a consequence, future studies may want to include higher incentives tied to high participant compliance than were provided in the current study (maximum of $120).

This study has several limitations. First, we recruited a sample of volunteers using social media platforms and participants had chronic pain and were currently using medical cannabis and opioids for pain management. Thus, the generalizability of our findings to other samples may be limited. Moreover, a substantial number of individuals who met inclusion criteria and consented to participate did not comply with the ID verification step. While verification of identity can be an important step to reduce the likelihood of enrolling fraudulent participants, some potentially legitimate individuals may be discouraged from participating due to privacy concerns (Heffner et al., 2021), which may further reduce sample generalizability. Lastly, the current study conducted frequent check-ins with participants throughout the study period to provide updates in compliance, which may have increased participant compliance and may limit the generalizability of our findings compared to studies that conduct less frequent check-ins. However, the current study also has strengths. Together with our previous work (Anderson Goodell et al., 2021), the current study demonstrates that EMA data collection is feasible in a diverse sample of individuals who suffer from high burden chronic pain and are using medical cannabis and opioids for pain management. Moreover, participant characteristics including age, sex, race/ethnicity, substance use, and chronic pain burden, were not associated with participant EMA compliance in multivariable regression analyses, which highlights that EMA data quality may be comparable across subgroups of participants. Finally, remote EMA data collection methods allowed us to conduct this study nationwide with a substantial number of participants.

In sum, the current study confirms the feasibility of using EMA methods to assess patterns of prescription opioids and medical cannabis among individuals experiencing chronic pain. Given the national burden of opioid use among individuals with chronic pain (Dahlhamer et al., 2021) and the high popularity of medical cannabis in this population (Boehnke et al., 2019), EMA data will be instrumental in investigating associations between cannabis and opioid use and chronic pain outcomes, which is a high priority for research.

## Funding

This work was supported by the 10.13039/100000026National Institute on Drug Abuse (T32 DA007292; R21 DA048175; UM1 DA059000). Jungin Joo was supported by a grant of the Korea Health Technology R&D Project through the Korea Health Industry Development Institute (KHIDI), funded by the Ministry of Health & Welfare, Republic of Korea (Grant Number: HI19C1328).

## Declaration of competing interest

The authors declare the following financial interests/personal relationships which may be considered as potential competing interests: In the past 3 years, KED has been paid as a consultant for Canopy, MindMed, and Cessation Therapeutics; received honoraria for advisory board work for Canopy Corporation and Beckley-Canopy; and received research and salary support from the National Institutes on Drug Abuse and Cure Addiction Now. PHF is on the advisory board for Ninnion Therapeutics. RV has received honoraria or consulting fees from Charlotte's Web, Jazz Pharmaceuticals, WebMD, Syqe Medical, and Mira1a Therapeutics.
